# SLE strikes the heart! A rare presentation of SLE myocarditis presenting as cardiogenic shock

**DOI:** 10.1186/s12872-021-02102-6

**Published:** 2021-06-13

**Authors:** Jaydeep J. Raval, Christina Rodriguez Ruiz, James Heywood, Jason J. Weiner

**Affiliations:** 1 Division of Rheumatology, Department of Internal Medicine, Naval Medical Center San Diego, 34800 Bob Wilson Drive, San Diego, CA 92134 USA; 2 Department of Cardiology, Scripps Clinic/Green Hospital, San Diego, CA USA; 3 9898 Genessee Ave, La Jolla, CA 92037 USA

**Keywords:** Systemic lupus erythematosus, SLE myocarditis, Myocarditis, Cardiogenic shock

## Abstract

**Background:**

Although systemic lupus erythematosus (SLE) can affect the cardiovascular system in many ways with diverse presentations, a severe cardiogenic shock secondary to SLE myocarditis is infrequently described in the medical literature. Variable presenting features of SLE myocarditis can also make the diagnosis challenging. This case report will allow learners to consider SLE myocarditis in the differential and appreciate the diagnostic uncertainty.

**Case presentation:**

A 20-year-old Filipino male presented with acute dyspnea, pleuritic chest pain, fevers, and diffuse rash after being diagnosed with SLE six months ago and treated with hydroxychloroquine. Labs were notable for leukopenia, non-nephrotic range proteinuria, elevated cardiac biomarkers, inflammatory markers, low complements, and serologies suggestive of active SLE. Broad-spectrum IV antibiotics and corticosteroids were initiated for sepsis and SLE activity. Blood cultures were positive for MSSA with likely skin source. An electrocardiogram showed diffuse ST-segment elevations without ischemic changes. CT chest demonstrated bilateral pleural and pericardial effusions with dense consolidations. Transthoracic and transesophageal echocardiogram demonstrated reduced left ventricular ejection fraction (LVEF) 45% with no valvular pathology suggestive of endocarditis. Although MSSA bacteremia resolved, the patient rapidly developed cardiopulmonary decline with a repeat echocardiogram demonstrating LVEF < 10%. A Cardiac MRI was a nondiagnostic study to elucidate an etiology of decompensation given inability to perform late gadolinium enhancement. Later, cardiac catheterization revealed normal cardiac output with non-obstructive coronary artery disease. As there was no clear etiology explaining his dramatic heart failure, endomyocardial biopsy was obtained demonstrating diffuse myofiber degeneration and inflammation. These pathological findings, in addition to skin biopsy demonstrating lichenoid dermatitis with a granular “full house” pattern was most consistent with SLE myocarditis. Furthermore, aggressive SLE-directed therapy demonstrated near full recovery of his heart failure.

**Conclusion:**

Although myocarditis during SLE flare is a well-described cardiac manifestation, progression to cardiogenic shock is infrequent and fatal. As such, SLE myocarditis should be promptly considered. Given the heterogenous presentation of SLE, combination of serologic evaluation, advanced imaging, and myocardial biopsies can be helpful when diagnostic uncertainty exists. Our case highlights diagnostic methods and clinical course of a de novo presentation of cardiogenic shock from SLE myocarditis, then rapid improvement.

## Background

Systemic lupus erythematosus (SLE) is an autoimmune disorder with a variable presentation affecting multiple organ systems. Typical presenting SLE features include constitutional symptoms, cutaneous involvement, alopecia, oral ulcers, arthritis, renal involvement, and common cardiac symptoms, including chest pain, dyspnea, palpitations, and lower extremity swelling [1]. Cardiac manifestations include conduction disturbances, ischemia, cardiomyopathy, pericarditis, and myocarditis; however, rapid progression leading to cardiogenic shock is rare [2]. Clinical myocarditis occurs in nearly 5–10% of SLE patients, although post-mortem studies suggest a higher prevalence of subclinical myocarditis than previously known [3]. Viral infections are the most common cause of myocarditis in the United States and other developed countries; however, other causes include alcohol, drugs, radiation, and autoimmune diseases with SLE and granulomatosis with polyangiitis (GPA) being the two most common causes [4]. There is no definitive consensus for optimal treatment, but the combination of immunosuppressive therapy and medical treatment of heart failure has shown favorable outcomes. Here we describe the clinical course of a 20-year-old male with a de novo presentation of cardiogenic shock secondary to SLE myocarditis with the diagnostic challenges we encountered.

## Case presentation

**Fig. 1:**
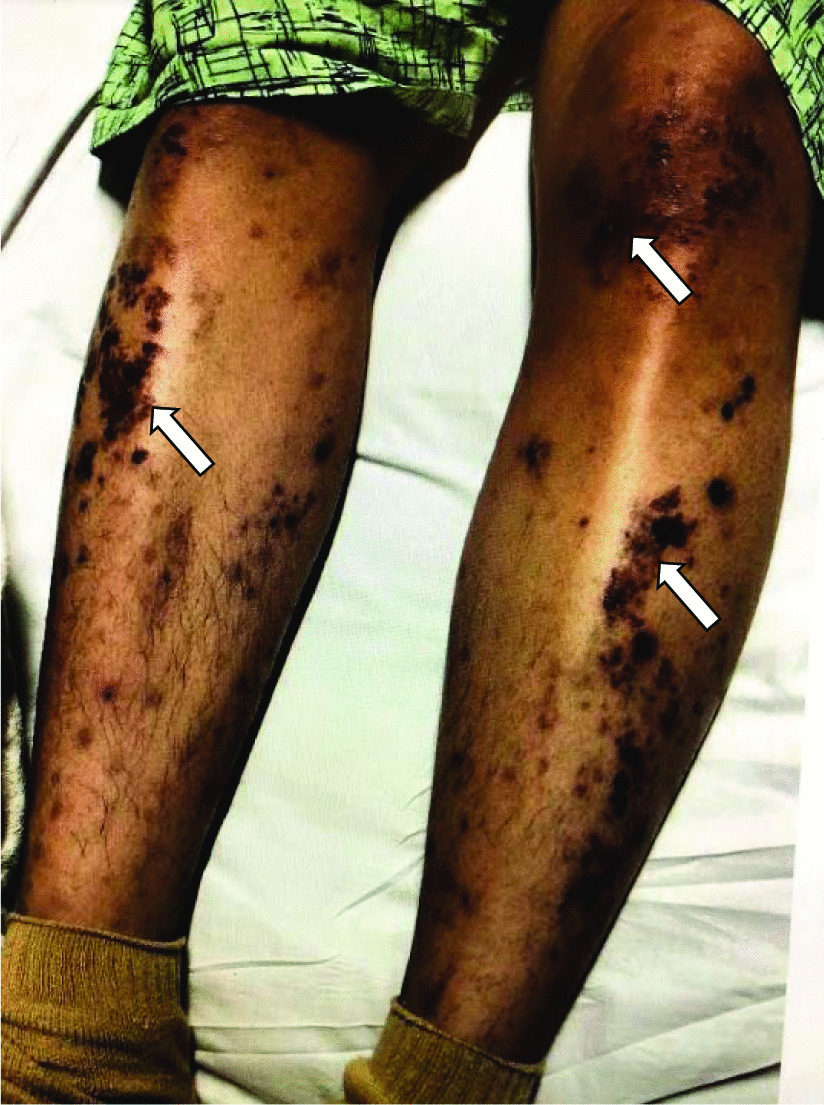
Skin examination on hospital day #1 revealing a diffuse hyperpigmented to violaceous rash with excoriated papules with overlying hemorrhagic crust and scales (white arrows), some coalescing into small plaques distributed on trunk, bilateral thigs, knees, anterior lower legs, and feet

**Fig. 2:**
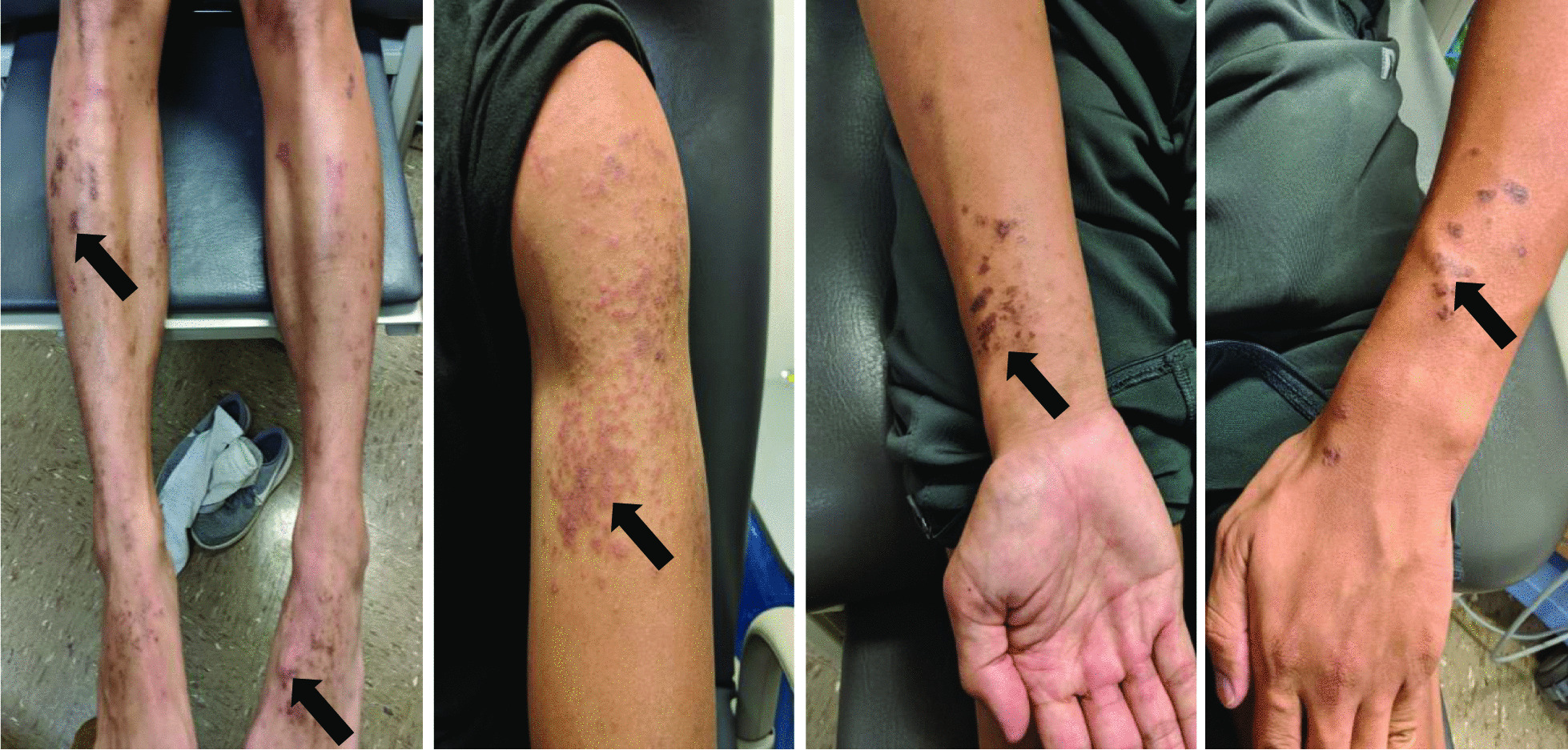
Skin examination during an outpatient clinic visit 6 months prior to his admission revealing a diffuse hyperpigmented rash with excoriated papules coalescing into plaques (black arrows) involving his bilateral upper and lower extremities and chest

**Fig. 3:**
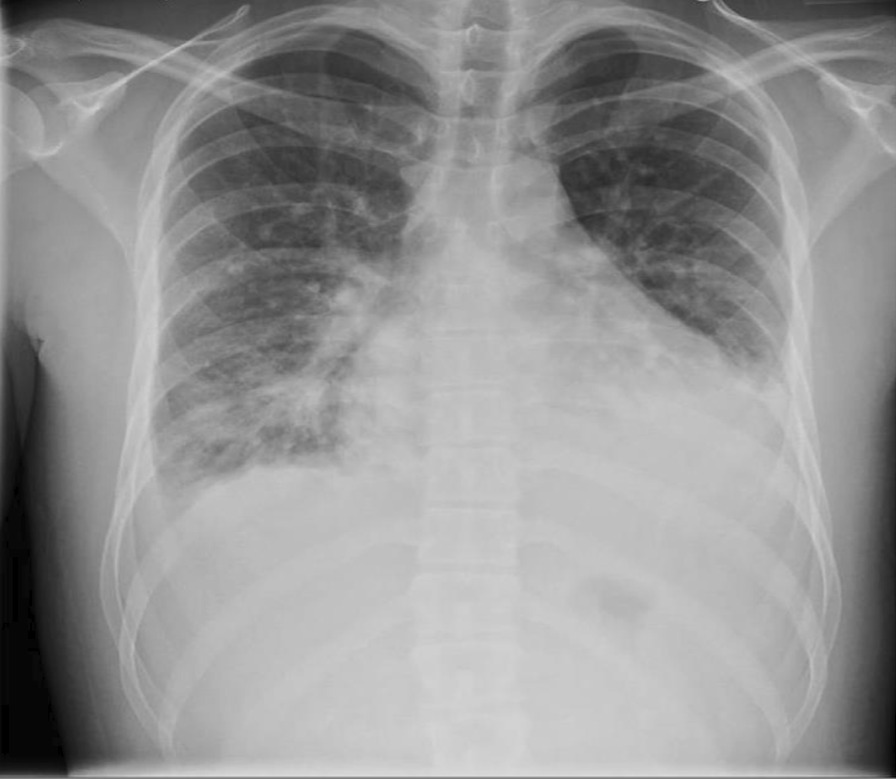
A portable chest x-ray demonstrating bilateral interstitial infiltrates with dense consolidations in lung bases, bilateral pleural effusions, and pericardial effusion

**Fig. 4:**
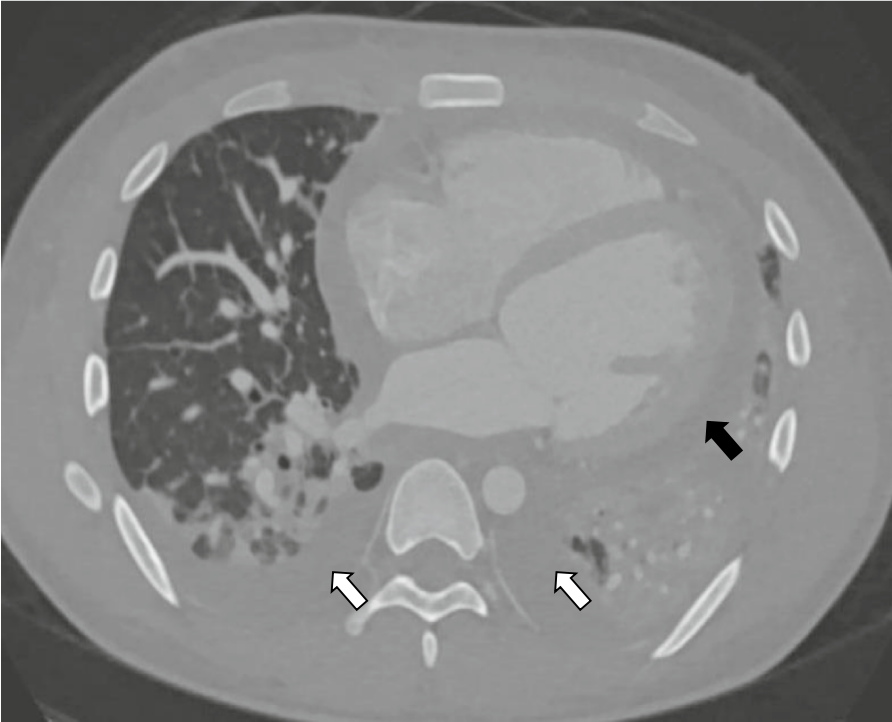
An axial view on computed tomography (CT) chest imaging obtained after the patient’s rapid cardiopulmonary decline demonstrating dense pulmonary consolidations, diffuse pulmonary nodules, and moderate pericardial (black arrow) and bilateral pleural effusions white arrows)

**Fig. 5:**
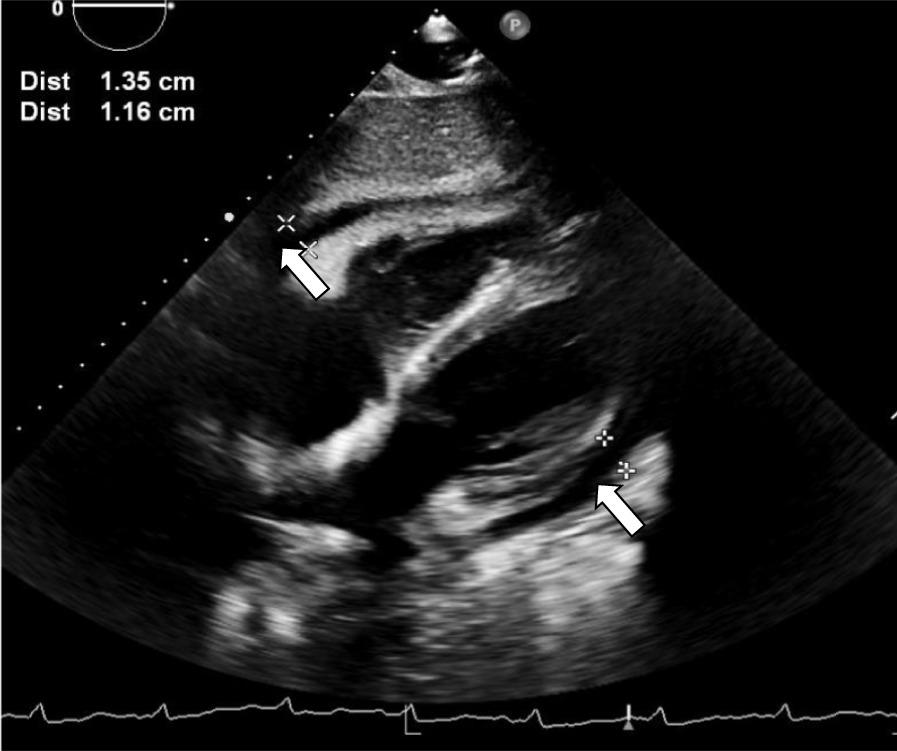
An apical four-chamber view on transthoracic echocardiogram obtained on the day of the patient’s cardiopulmonary decline (HD #6) demonstrating a moderate-sized pericardial effusion (white arrows)

**Fig. 6:**
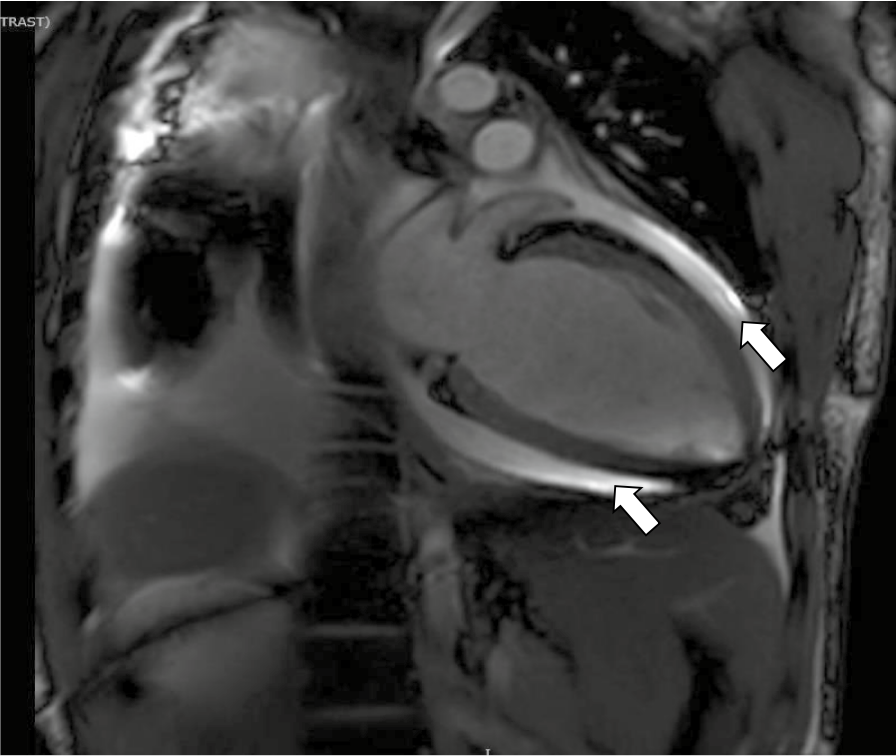
A Cardiac MRI, T-2 weighted axial image of diastolic phase with late gadolinium enhancement sequence, demonstrating left ventricular dilation and large bilateral pleural effusions and pericardial effusions (white arrows). Myocardial tissue characterization with late gadolinium hyperenhancement was unable to be performed due to patient’s inability to lie recumbent and tachypnea

Our patient is a 20-year-old Filipino male diagnosed with SLE 6 months prior and was started on hydroxychloroquine. The patient presented to the emergency department after sudden onset of dyspnea, pleuritic chest pain, generalized fatigue, dry cough, fevers and chronic progressive rash. His review of systems was negative for sick contacts, recent travel, engagement in high-risk sexual activity, intravenous drug use, was a lifetime nonsmoker, and did not consume alcohol. On evaluation, vitals were notable for normal blood pressure (118/72 mmHg), tachycardia (118 beats per minute), tachypnea (respiratory rate 22 breaths per minute), and fever (100.8 °F). His cardiovascular exam was unremarkable for murmurs, elevated jugular venous pressure, splinter hemorrhages, or lower extremity edema. No cyanosis, clubbing, scleral icterus, organomegaly, or lymphadenopathy was appreciated. The skin examination showed no oral ulcers or alopecia, but demonstrated hyperpigmented to violaceous, scaly plaques with excoriated papules involving the bilateral extremities, back, and chest worsened as shown in Fig. 1. A prior skin punch biopsy of a similar rash (Fig. 2) showed lichenoid dermatitis with features of interface changes and chronic inflammation suggestive of cutaneous SLE. This rash had improved from his index presentation following treatment with hydroxychloroquine, though not fully resolved. Laboratory data on admission revealed the following: leukopenia, anemia, and non-nephrotic range proteinuria without active urinary sediment on urine microscopy. There was evidence of acute myocardial injury with elevated cardiac biomarkers along with an elevated N-terminal pro-brain natriuretic peptide. Additionally, inflammatory biomarkers were elevated with hypocomplementemia, positive antinuclear antibody (ANA) with anti-Smith, ribonucleoprotein, chromatin, SS-A, double-stranded DNA, perinuclear anti-neutrophil cytoplasmic antibodies (pANCA), and myeloperoxidase positivity. Comprehensive laboratory evaluation for additional workup and the reference ranges is shown in Table 1. His electrocardiogram and telemetry monitoring demonstrated normal sinus rhythm with diffuse < 1 mm ST-segment elevations in leads without any ischemic changes. Initial chest x-ray showed no acute cardiopulmonary process. Further infectious evaluation demonstrated positive blood cultures for methicillin-sensitive *Staphylococcus aureus* (MSSA) with a likely skin source from aggressively scratching a purulent lesion on the right anterior thigh. Initial transthoracic echocardiogram showed normal LVEF with all other normal parameters. Given concerns for sepsis secondary to presumed cellulitis, in the setting of SLE flare, he was treated with broad spectrum intravenous (IV) antibiotics and corticosteroids (methylprednisolone 1 g/kg for three days) before his transfer to our institution. Given concerns for endocarditis with persistent fevers on arrival, repeat transthoracic and transesophageal echocardiograms demonstrated mildly reduced LVEF 45% with no valvular pathology. MSSA bacteremia resolved within 72 h of intravenous antibiotics (cefazolin 2 g IV every 8 h), but the patient later developed a rapid cardiopulmonary decline on hospital day #5 with worsening dyspnea, pleuritic chest pain, and hemoptysis. A repeat chest x-ray (Fig. 3) and CT chest (Fig. 4) were obtained demonstrating dense pulmonary bilateral pleural effusions with dense consolidations, pulmonary nodules, possible pulmonary emboli within the left subsegmental pulmonary artery branches, and pericardial effusions. A repeat transthoracic echocardiogram demonstrated a severely reduced LVEF 25%, moderate global hypokinesis, and small pericardial effusion without evidence of tamponade (Fig. 5). Other abnormal findings included elevated right atrial pressures (15 mmHg) and mild pulmonary hypertension (43 mmHg) with a normal left atrial pressure, right ventricular systolic function, left ventricular diastolic function, and valvular function. Given clearance of bacteremia with IV antibiotics, cardiogenic shock was favored over septic shock. Intravenous vasopressors (norepinephrine, 0.4mcg/kg/min IV infusion) for shock, diuretics, amiodarone drip for atrial fibrillation with a rapid ventricular response, heparin drip for submassive pulmonary emboli, high-dose corticosteroids (solumedrol 500 mg IV every 12 h) and hydroxychloroquine 400 mg daily for SLE related co-activity were initiated. A cardiac MRI (CMR) was subsequently pursued to clarify the cause of myocarditis, which demonstrated a LVEF of 25% and moderate global hypokinesis with worsening pericardial effusions (Fig. 6). Unfortunately, the patient’s poor respiratory reserve and tachycardia limited our ability to perform late gadolinium enhancement. With his continued clinical decline potentially requiring escalating hemodynamic support, he was transferred to a medical center with advanced heart failure treatment capabilities.

**Table 1:** Laboratory work-up performed during hospitalization

Laboratory test index	Results	Reference ranges
Total WBC count	3.7 × 10^3^ mcL (L)	4.0–10.5 × 10^3^ mcL
Differential		
Neutrophils	85.5% (H)	40.0–80.0%
Lymphocytes	7.9% (L)	15.0–45.0%
Absolute lymphocyte count	0.6 × 10^3^ mcL (L)	1.0–4.5 × 10^3^ mcL
Hb	10.4 g/dL (L)	13.8–17.0 g/dL
MCV	80.8 fL (L)	82.0–99.0 fL
Platelets	188 × 10^3^ mcL	150–450 × 10^3^ mcL
AST	94 U/L (H)	12–39 U/L
ALT	67 U/L (H)	17–63 U/L
LDH	239 U/L (H)	135–225 U/L
Troponin T	0.053 ng/mL (H) Peaked at 0.94 ng/mL	< 0.010 ng/mL
NT-proBNP	2210 pg/mL (H)	< 125 pg/mL
ESR	87 mm/h (H)	0–10 mm/h
CRP	12.21 mg/dL (H)	< 0.50 mg/dL
Haptoglobin	175 mg/dL	30–200 mg/dL
C3	23 mg/dL (L)	90–180 mg/dL
C4	3 mg/dL (L)	10–40 mg/dL
Rheumatoid factor	11 IU/mL	< 13 IU/mL
ANA screen, IFA, with reflex to titer and pattern	Positive > 1:80; homogenous pattern	< 1:40
Double-stranded DNA Ab	> 300 IU/mL (H)	0–9 IU/mL
Anti-Smith Ab	Positive	Negative
Anti-RNP Ab	Positive	Negative
Anti-ribosomal-P Ab	Positive	Negative
Anti-chromatin Ab	Positive	Negative
Anti-SS-A (Ro) Ab	Positive	Negative
Anti-SS-B (La) Ab	Negative	Negative
Jo-1 extractable nuclear Ab	Negative	Negative
Anti-centromere Ab	Negative	Negative
SCL-70 extractable nuclear Ab	Negative	Negative
ANCA panel		
Neutrophil cytoplasmic Ab cytoplasmic	Negative	Negative
Neutrophil cytoplasmic Ab perinuclear	1:2560	Negative
Myeloperoxidase Ab	51 AU/mL	0–19.0 AU/mL
Proteinase 3 Ab	< 3.5 AU/mL	0–19.0 AU/mL
Antiphospholipid Ab	153 mg/dL	150 – 250 mg/dL
Quantitative immunoglobulin panel		
IgM	35 mg/dL	40–230 mg/dL
IgA	231 mg/dL	70–400 mg/dL
IgG	1103 mg/dL	700–1600 mg/dL
IgG subclass 4	29 mg/dL	2–96 mg/dL
Lupus anticoagulant	2 AU/mL	0–40 AU/mL
Anti-cardiolipin Ab, IgG	< 9 GPL/mL	0–14 GPL/mL
Anti-cardiolipin Ab, IgM	< 9 MPL/mL	0–12 MPL/mL
Beta-2 glycoprotein 1 Ab, IgG	< 9 GPI IgG units	0–20 GPI IgG units
Beta-2 glycoprotein 1 Ab, IgM	< 9 GPI IgM units	0–32 GPI IgM units
Beta-2 glycoprotein 1 Ab, IgA	< 9 GPI IgA units	0–25 GPI IgA units
Streptolysin O Ab	22 IU/mL	< 20–200 IU/mL
DNase B Ab streptococcal	< 78 U/mL	0–120 U/mL
Angiotensin converting enzyme	< 40 nmol/mL/min	0–40 nmol/mL/min
Urine protein/creatinine ratio	663 mg/24 h	< 0.2 mg/24 Hr
Urine microscopy	No active urine sediment	Negative
Kappa/Lambda light chain	< 0.26 mg/L	0.26–1.25 mg/L
Blood cultures with sensitivities		
HD #1 2/2 MSSA (sensitive to cefazolin, ampicillin/sulbactam, oxacillin)	Positive	Negative
HD #2	No growth	Negative
HD #3	1/4 Staph epidermitidis (contaminant)	Negative
HD #5	No growth	Negative
HD #6	No growth	Negative
HD #7	No growth	Negative
Respiratory cultures		
HD #6	GPCs in pairs/chains, GNRs, and yeast (with squamous epithelial cells)-respiratory flora	Negative
Expanded respiratory viral panel	Negative	Negative
Influenza virus A RNA		
Influenza virus B RNA		
Influenza virus A Hemagglutinin H1 RNA		
Influenza virus A Hemagglutinin H3 RNA		
Resp syncytial virus A PCR		
Resp syncytial virus B PCR		
Parainfluenza virus 1 RNA		
Parainfluenza virus 2 RNA		
Parainfluenza virus 3 RNA		
Parainfluenza virus 4 RNA		
Adenovirus DNA		
Human metapneumovirus RNA		
Rhinovirus RNA		
Bordetella pertussis DNA		
Bordetella holmseii DNA		
Bord Parapert/Bronchiseptica		
Hepatitis viral panel		
Hepatitis A virus Ab	Reactive	Reactive
Hepatitis A IgM	Non-reactive	Non-reactive
Hepatitis B virus core Ab	Non-reactive	Non-reactive
Hepatitis B virus core Ab IgM	Non-reactive	Non-reactive
Hepatitis B virus surface Ab	Reactive	Reactive
Hepatitis B virus surface Ag	Non-reactive	Non-reactive
Hepatitis C virus Ab	Non-reactive	Non-reactive
Epstein–Barr virus capsid Ab IgG	Positive (H)	Negative
Epstein–Barr virus capsid Ab IgM	Negative	Negative
Cytomegalovirus Ab IgG	Positive (H)	Negative
Cytomegalovirus Ab IgM	Negative	Negative
Coxsackie A IgG	Negative	Negative
Coxsackie A IgM		
HIV 4th gen	Negative	Negative
HTLV-1+2 Ab	Negative	Negative
*Treponema pallidum* Ab	Non-reactive	Non-reactive
1,3 Beta-D Glucan	< 31 pg/mL	< 80 pg/mL
*Coccidioides immitis* Ab IgG	Negative	Negative
*Coccidioides immitis* Ab IgM	Negative	Negative
Cryptococcus sp. Ag	Negative	Negative
Aspergillus galactomannan Ag	0.04 ng/mL	0.00–0.49 ng/mL
Histoplasma urinary antigen	Negative	Negative

WBC, white blood count; Hb, hemoglobin, MCV, mean corpuscular volume; AST, aspartate aminotransferase; ALT, alanine aminotransferase; LDH, lactate dehydrogenase; NT-proBNP, N-terminal (NT)-pro hormone BNP; ESR, erythrocyte sedimentation rate; CRP, C-reactive protein; C3, complement c3; C4, complement c4, ANA, antinuclear antibody; IFA, Indirect Immunofluorescence Assay; RNP, ribonucleoprotein; ANCA, antineutrophil cytoplasmic antibodies; MSSA, Methicillin-sensitive Staph aureus; GPCs, gram positive cocci; GNR, gram negative rods; HIV, human immunodeficiency virus; HTLV, human T-lymphotropic virus

**Fig. 7:**
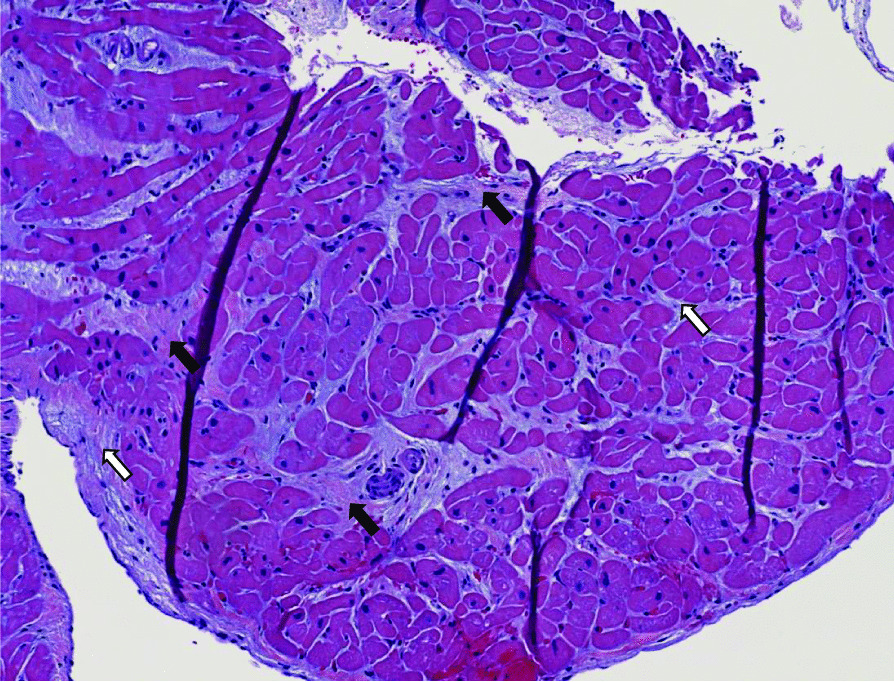
Histology of endomyocardial biopsy from the right ventricle with hematoxylin and eosin stain depicting normal myocardium with interstitial edema (black arrows) with early fibrosis highlighted by trichrome stain (white arrows)

**Fig. 8:**
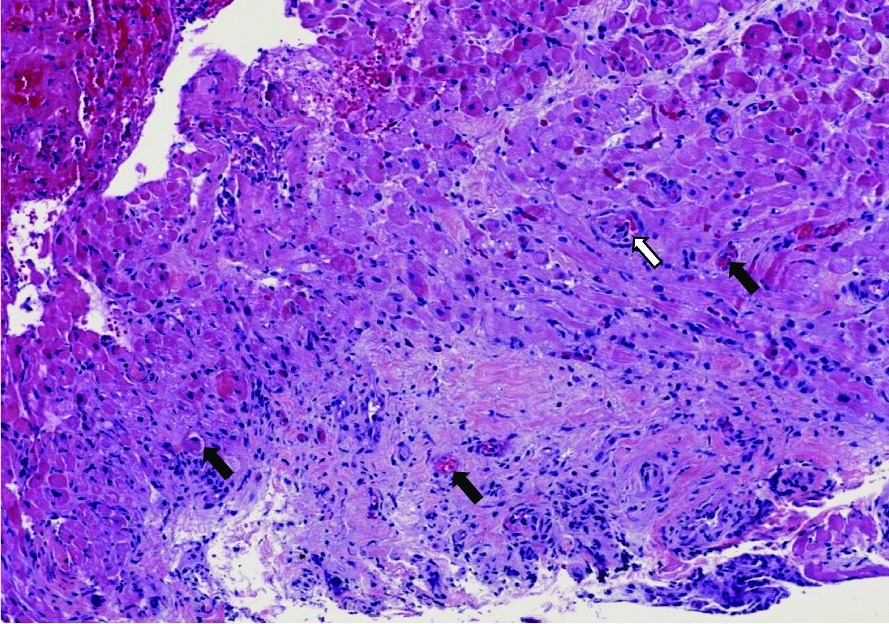
Histology of endomyocardial biopsy with hematoxylin and eosin stain depicting an abnormal myocardium region with diffuse myofiber degeneration with significant architectural disarray, extensive infiltration of macrophages (CD68 positive) admixed with a few lymphocytes (CD45 immunostaining), neoangiogenesis (black arrows), and perivascular inflammation. Specifically, no granulomas, giant cells, or cytoplasmic vacuolization are visualized here. The spectrum of morphologic findings was highly suggestive of SLE myocarditis

**Fig. 9:**
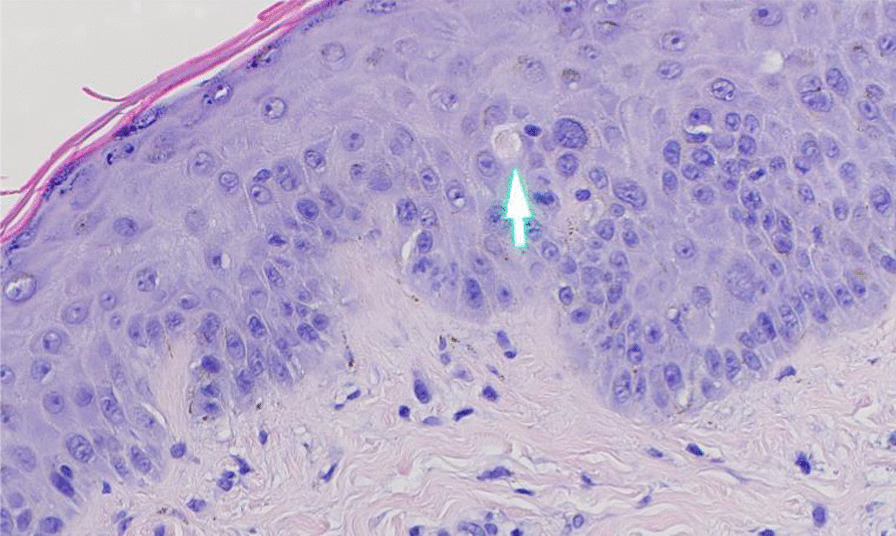
A histology section of a skin punch biopsy with hematoxylin and eosin stain demonstrating superficial lymphocytic and neutrophilic infiltration within epidermis and dermis, vacuolization of the basal cell layer and rare apoptotic keratinocytes (white arrow) seen in lichenoid (interface) dermatitis compatible with cutaneous lupus erythematosus

The differential etiologies leading to a precipitous cardiopulmonary decline in our SLE patient included infectious causes, autoimmune causes including an overlapping ANCA vasculitis, ischemic etiologies, septic cardiomyopathy, metabolic, and other toxic causes such as hydroxychloroquine-induced cardiomyopathy. An extensive infectious work-up included negative viral and fungal serologies, negative surveillance blood cultures, and respiratory cultures as listed in Table 1. The lack of arthritis, thrombocytopenia, and alveolar hemorrhage with ongoing systemic features better fit SLE, than overlapping ANCA vasculitis. In the absence of any other etiology to explain such a dramatic decline, a diagnosis of SLE myocarditis was most likely. Echocardiogram was repeated on hospital day #7 at the outside institution, which showed interval worsening of LVEF 10% with global hypokinesis, moderate concentric hypertrophy (LV mass index: 143.2 g/m^2^), and moderate pericardial effusion. Other echocardiogram parameters were normal. In addition to aforementioned therapy for SLE, medical management of his heart failure was instituted with angiotensin receptor neprilysin inhibitor (sacubitril/valsartan 24–26 mg twice daily), b-blocker (carvedilol 6.25 mg twice daily), digoxin 0.125 mg daily, amiodarone 200 mg daily, and mineralocorticoid receptor antagonist (spironolactone 12.5 mg daily). Following a relative improvement in hemodynamics, an endomyocardial biopsy was performed on hospital day #10 to elucidate the etiology of decompensation. Concurrently, a right and left heart cardiac catheterization and coronary angiogram were performed for a comprehensive cardiac workup, which revealed normal right and left-sided filling pressures, no evidence of pulmonary hypertension, normal cardiac output and index, and non-obstructive coronary artery disease. Later, his endomyocardial biopsy demonstrated extensive myocardial degeneration with architectural disarray, neoangiogenesis, and perivascular inflammation (Figs. 7, 8) suggestive of myocarditis. Notably, there were no granulomas, giant cells, or cytoplasmic vacuolization. Additionally, a repeat skin biopsy of a thigh lesion performed earlier revealed lichenoid interface dermatitis with vacuolization, and chronic inflammation (Fig. 9) with diffuse immunofluorescence demonstrated a granular deposition of IgA, IgG, C3, and fibrin (full house pattern) at the dermoepidermal junction. These pathological results of skin and endomyocardial biopsies further supported systemic SLE. The patient rapidly improved as his echocardiogram on the day of discharge (hospital day #12) showed improved LVEF 45% with improving pericardial effusion and normal left ventricular mass, and wall thickness. He was weaned off vasopressors and discharged on medical therapy for heart failure, oral prednisone 20 mg daily, hydroxychloroquine 400 mg daily, trimethoprim-sulfamethoxazole double-strength 800/160 mg twice daily for cellulitis, and cefazolin 2 mg IV daily MSSA bacteremia.

At one month follow-up in rheumatology clinic, the patient was started on additional steroid-sparing therapy with monthly infusions of cyclophosphamide (750 mg/m^2^) given systemic SLE with cutaneous manifestations. After completing four cycles of cyclophosphamide and steroid taper, cardiac MRI was repeated after 3 months to assess interval activity. His severely impaired LVEF and pericardial effusions were resolved with no evidence of myocardial scarring or evidence suggesting active SLE on LGE and edema sequences. Echocardiogram after 6 months also showed normal LVEF > 55%, left ventricular diastolic function and mass, wall motion, and global longitudinal strain similar to his baseline findings. Laboratory evaluation showed a significant improvement in his inflammatory markers and cytopenias (leukopenia and anemia) shown in Table 2. His SLE is currently treated with mycophenolate mofetil 100 mg bid (transitioned from cyclophosphamide given favorable side effect profile with potentially long-term therapy) for maintenance therapy with a prednisone taper (60 mg daily for three weeks, taper by 10 mg every two weeks down to 10 mg daily), hydroxychloroquine 400 mg daily, and aforementioned heart failure guideline-directed medical therapy. His functional status is classified as New York Heart Association (NYHA) class I with continued clinical recovery (Table 3).

**Table 2:** Laboratory work-up performed at 3-month outpatient rheumatology follow-up

Laboratory test index	Results	Reference ranges
Total WBC count	7.9 × 10^3^ mcL	4.0–10.5 × 10^3^ mcL
Neutrophils	55%	40.0–80.0%
Lymphocytes	29%	15.0–45.0%
Absolute lymphocyte count	2.29 × 10^3^ mcL	1.0–4.5 × 10^3^ mcL
Hb	15.1 g/dL	13.8–17.0 g/dL
Platelets	239 × 10^3^ mcL	150–450 × 10^3^ mcL
C3	104 mg/dL	90–180 mg/dL
C4	21 mg/dL	10–40 mg/dL
DNA double stranded Ab	69 IU/mL	0–9 IU/mL
Urine protein/creatinine ratio	120 mg/24 h	< 0.2 mg/24 h

WBC, white blood count; Hb, hemoglobin, C3, complement c3; C4, complement c4

**Table 3:** Timeline sequence of patient’s critical clinical events

September 2019	Patient was diagnosed with SLE based on EULAR/ACR criteria and started on hydroxychloroquine after presenting with arthritis, alopecia, rash, and serologic evaluation. Findings of skin biopsy were suggestive of cutaneous SLE
3.7.20. HD#1	Patient presented to the emergency department of an outside facility with pleuritic chest pain, fatigue, dyspnea, fevers, and worsening rash. Laboratory workup was suggestive of SLE flare and sepsis likely secondary to MSSA bacteremia with a skin source. Broad-spectrum antibiotics and pulse dose corticosteroids (solumedrol 1 mg/kg for three days) initiated. Initial transthoracic echocardiogram demonstrated normal LVEF > 55% with no valvular pathology
3.9.20. HD #3	Patient was transferred to our facility per his insurance eligibility in a hemodynamically stable condition. Intravenous cefazolin was continued for MSSA bacteremia. Repeat blood and surveillance blood cultures were negative. TEE showed LVEF of > 45% but no evidence of valvular pathology
3.12.21. HD #6	With recurrence of dyspnea and pleuritic chest pain and hemodynamic instability, the patient was transferred to the ICU. A CT chest with pulmonary embolism protocol demonstrated possible pulmonary embolism, bilateral pleural effusions, dense consolidations with pulmonary nodules, and pericardial effusions. Serial TTE on the same day showed interval decline in LVEF of 45% down to 25% and worsening pericardial effusions. Treatment with heparin drip for possible pulmonary emboli, broad-spectrum IV antibiotics for possible septic emboli, solumedrol 500 mg IV q12h for severe SLE co-activity was started, hydroxychloroquine 400 mg daily was continued, and vasopressors for cardiogenic shock were initiated
3.13.20. HD #7	Guideline directed medical therapy for heart failure was escalated. A cardiac MRI demonstrated a reduced LVEF 25% and moderate global hypokinesis with worsening pericardial effusions; however, this limited study did not reveal an etiology explain his decompensation. The patient was transferred to an institution with capabilities to treat advanced heart failure
3.14.20. HD #8	A repeat TTE showed markedly decreased LVEF 10% with severe global hypokinesis. Intravenous steroids and guideline-directed heart failure therapy was continued
3.17.20. HD #11	In efforts to further explain cause of decompensation and unrevealing CMR, endomyocardial biopsy was performed along with a concurrent Left and right heart catheterization. Coronary angiogram showed normal cardiac output, cardiac index, left and right-sided filling pressures, and non-obstructive coronary arteries
3.18.20. HD #12	With improving hemodynamics on SLE and heart failure- targeted therapy, the patient was weaned off vasopressors, and repeat TTE showed mildly dilated left ventricle with improved LVEF 45% from previously 10%. The patient was discharged on oral medical therapy of heart failure, hydroxychloroquine, and prednisone for SLE, and IV antibiotics for MSSA bacteremia in a stable condition
Late March–April 2020	The patient was transitioned to four cycles of steroid-sparing therapy with Cyclophosphamide and later transitioned to mycophenolate mofetil due to favorable side effect risk profile. Hydroxychloroquine, and heart failure therapy were continued
September–November 2020	A repeat Cardiac MRI and TTE to assess interval SLE activity showed normal left ventricular systolic function and no evidence of myocardial scarring or pericardial effusions. Patient returned to normal baseline functional status

## Discussion

Systemic lupus erythematosus is an independent risk factor for cardiovascular disease with a tenfold increased risk of complications as cardiac involvement may indicate disease severity [5]. Any cardiac structural components can be affected and may manifest as myocarditis, pericarditis, noninfectious (Liebman–Sacks) endocarditis, vasculitis, myocardial infarction, and heart failure [5, 6]. The underlying chronic inflammatory state of SLE inciting an immunologic injury from immune-complex deposition and complement activation has been postulated as the underlying mechanism of pathogenesis for increased atherogenesis, autoantibody production, endothelial dysfunction noted on postmortem myocardial biopsies [7]. We suspect a similar immunologic mechanism that contributed to ramped disease course leading to a dramatic cardiopulmonary decline on our patient. The prevalence of clinical SLE Myocarditis is estimated to be 5–10%, although recent studies suggest a higher prevalence of subclinical myocarditis as supported by evidence of acute myocardial injury in 37% of SLE patients from a recent study [3]. Although many studies suggest an increased risk of mortality for any clinically active SLE myocarditis, the prognostic implications of subclinical myocardial injury are unknown [3]. Despite generally favorable outcomes in SLE myocarditis, severe cardiac impairment on index presentation is associated with significant morbidity and mortality [1] (Table 4).

**Table 4:** Depiction of the patient’s LVEF during his clinical course

Hospital day	Left ventricular ejection fraction (%)
1	60
6	45
7	25
8	10
12	45
6-month follow up	60

The diagnosis of SLE is established according to the 2019 classification criteria of European League Against Rheumatism (EULAR) and American College of Rheumatologic (ACR) [1]. Although heterogenous presentation of SLE myocarditis can pose diagnostic challenges. Our patient was initially diagnosed with SLE according to the EULAR/ACR classification (positive ANA, positive anti-Smith/RNP/dsDNA/chromatin/Ro/Ribosomal-P antibodies, low complements, leukopenia, positive agglutinin test without hemolysis, alopecia, photosensitivity, malar rash, and arthritis) and concordant findings of skin biopsy suggestive of cutaneous SLE. On admission, the laboratory evaluation suggested ramped SLE activity, though it was uncertain whether it was the primary driver underlying his cardiopulmonary decompensation in the context of concurrent sepsis. When a clinical dilemma exists, a combination of serologic evaluation and advanced cardiac imaging modalities such as conventional echocardiography and cardiac magnetic resonance imaging can be helpful in the diagnostic evaluation. Increased gadolinium enhancement on CMR can help characterize the spectrum of inflammatory changes within the myocardium and speckle tracking features within echocardiography provide prognostic implications in evaluating both clinical and subclinical myocarditis [8, 9]. Unfortunately, CMR feature was precluded by the patient’s poor respiratory effort and tachycardia during the initial study. However, repeat CMR obtained at 6-months to evaluate for any residual effects of SLE myocarditis showed no evidence of myocardial scarring or SLE activity on LGE and edema sequences. Endomyocardial biopsy is the gold standard test when diagnostic uncertainly exists in ruling out other alternative causes; however, it’s not routinely performed given its potential for procedural-related risks and sampling error that stems from patchy tissue involvement leading non-diagnostic results [7, 10, 13–15]. After a nondiagnostic CMR, endomyocardial biopsy was pursued which showed typical histopathologic features of myocarditis. These include regions of architectural disarray from infiltration of inflammatory cells and hypereosinophilic fibers leading to myocardial edema and necrosis as depicted in our patient’s endomyocardial biopsy shown in Figs. 7, 8 [7, 11].

Differentiating between the etiologies of myocarditis can be a clinical dilemma, as evident in our patient. Other etiologies, including metabolic, toxic, ischemic, infectious, and other autoimmune causes, were excluded by a negative laboratory workup and nonobstructive coronaries on coronary angiography. Septic cardiomyopathy was initially considered with MSSA bacteremia and initial concerns for potential septic emboli and developing abscesses on CT imaging, although deemed less likely given negative respiratory and surveillance blood cultures from treatment with IV antibiotics, lack of persistent evidence on serial imaging, and clinical improvement with SLE-targeted therapy [4]. Further unremarkable infectious workup included viral serologies for respiratory viruses, hepatitis panel, HIV, EBV, CMV, and fungal serologies for coccidioidomycosis, blastomycosis, cryptococcus, and histoplasmosis. Positive CMV IgM serology was less likely to represent an invasive CMV disease as large pentamers of IgM frequently cause false positives on ELISA testing. Hydroxychloroquine cardiomyopathy was not contributory given the absence of cytoplasmic vacuolization on cardiac biopsy and short duration of exposure to hydroxychloroquine, which typically occurs after years [12]. In the context of positive p-ANCA and MPO, an overlap of ANCA-associated vasculitis with SLE was considered, but less likely in the absence of any other systemic overlapping features, which typically include arthritis, renal injury with active urinary sediment, thrombocytopenia, or alveolar hemorrhage [1]. Drug-induced myocarditis was less likely in the absence of exposure to commonly-associated drugs such as hydralazine, procainamide, sulfa, isoniazid, and illicit substances such as cocaine [1]. Results of an extensive diagnostic evaluation featuring serologic profile (positive ANA, anti-dsDNA), radiographic evidence of serositis (pleural and pericardial effusions), and clinical presentation (chest pain, constitutional symptoms, and rash), and dramatic improvement with immunosuppressive therapy suggested SLE be the culprit of cardiogenic shock in our patient.

**Fig. 10:**
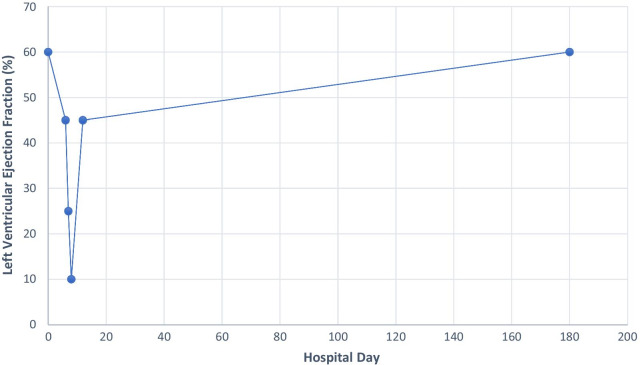
A line graph depicting the patient’s LVEF during his clinical course

There is a paucity of data on optimal treatment strategies for cardiogenic shock secondary to SLE myocarditis as current guidance is based upon consensus driven by case studies and observational data. Immunosuppression, usually with high-dose steroids is the cornerstone of treatment, although cyclophosphamide, azathioprine, mycophenolate, and intravenous immunoglobulin can be alternative options to control disease activity based on review of recent case reports described in literature [2, 3]. Cyclophosphamide was utilized as an agent for steroid-sparing therapy given that it has been commonly used in SLE nephritis, severe cutaneous SLE, and other reported cases of myocarditis due to its favorable side effect profile and his Asian ethnicity [3]. Treatment in refractory cases involves disease-modifying novel biologic agents such as canakinumab, belimumab, and rituximab have also shown a trend towards positive clinical outcomes [6]. Despite early data suggesting a trend towards positive outcomes with immunosuppressive therapy, further studies aimed to investigate definitive treatment are needed for optimal management of SLE myocarditis. The treatment of severe cardiomyopathy involves guideline-directed medical therapy (ACE or ARB or ARNI, BB, MRA, isosorbide dinitrate-hydralazine, SGLT-2 inhibitors) for heart failure; however, intractable cases may involve utilization of cardiac resynchronization therapy (CRT), intraaortic balloon pump (IABP), mechanical circulatory support device (MCS), extracorporeal membrane oxygenation (ECMO), to augment left ventricular function with cardiac transplantation reserved as a last resort [6]. Fortunately, our patient’s severely compromised cardiac function rapidly improved with the combination of immunosuppressive therapy and medical therapy for heart failure sparing utilization of advanced mechanical support (Fig. 10).

## Conclusion

Despite its low prevalence, SLE myocarditis should be promptly considered in SLE patients presenting with new-onset heart failure given high morbidity and mortality rates. Early diagnosis and treatment with immunosuppressive therapy and heart failure medical management may lead to positive clinical outcomes. This case highlights a rare index cardiac presentation of SLE myocarditis leading to cardiogenic shock with rapid clinical recovery on immunosuppressive therapy and supportive care.

## Data Availability

Not applicable.

## Abbreviations

SLE: Systemic lupus erythematosus
ANA: Antinuclear antibody
RNP: Ribonucleoprotein
ENA: Extractable nuclear antigen antibodies panel
dsDNA: Double stranded DNA
GPA: Granulomatosis with polyangiitis
pANCA: Perinuclear anti-neutrophil cytoplasmic antibodies
MPO: Myeloperoxidase
MSSA: Methicillin-sensitive *Staphylococcus aureus*
PR-3: Proteinase 3
LVEF: Left ventricular ejection fraction
NT pro-BNP: N-terminal pro-brain natriuretic peptide
NYHA: New York Heart Association classification of heart failure
